# Striving for Success but at What Cost? Subject-Specific Achievement Goal Orientation Profiles, Perceived Cost, and Academic Well-Being

**DOI:** 10.3389/fpsyg.2020.557445

**Published:** 2020-09-29

**Authors:** Heta Tuominen, Henriikka Juntunen, Markku Niemivirta

**Affiliations:** ^1^Turku Institute for Advanced Studies & Department of Teacher Education, University of Turku, Turku, Finland; ^2^Department of Education, University of Helsinki, Helsinki, Finland; ^3^School of Applied Educational Science and Teacher Education, University of Eastern Finland, Joensuu, Finland

**Keywords:** motivation, achievement goal orientation, expectancy-value theory, cost, school engagement, school burnout, mathematics, English

## Abstract

Most studies utilizing a person-oriented approach to investigating students’ achievement goal orientation profiles have been domain-general or focused on a single domain (usually mathematics), thus excluding the possibility of identifying distinct subject-specific motivational profiles. In this study, we looked into this by examining upper secondary school students’ subject-specific achievement goal orientation profiles simultaneously in mathematics and English. As distinct profiles might contribute to how students invest time and effort in studying, we also examined differences in perceived subject-specific cost (i.e., effort required, emotional cost, opportunity cost) among students with different profiles and how this was linked with students’ more general academic well-being (i.e., school engagement, burnout). The 434 Finnish general upper secondary school students participating in the study were classified based on their achievement goal orientations in the two subjects using latent profile analysis, and the predictions of the latent profile on distal outcomes (i.e., measures of cost and academic well-being) were examined within the mixture model. Five divergent achievement goal orientation profiles were identified: *indifferent* (29%), *success-oriented* (26%), *mastery-oriented* (25%), *English-oriented*, *math-avoidant* (14%), and *avoidance-oriented* (6%). The English-oriented, math-avoidant students showed the most distinct domain-specificity in their profile but, in general, profiles indicated more cross-domain generality than specificity. Overall, mastery-oriented students showed the most adaptive academic well-being, while avoidance-oriented students were the least engaged. Success-oriented students were characterized by high multiple goals in both subjects, elevated costs, and high scores on both positive (engagement) and negative (burnout) well-being indicators. The English-oriented, math-avoidant students perceived studying math as costly. The findings suggest that addressing students’ achievement motivation in different subjects may be useful for recognizing factors endangering or fostering student learning and well-being.

## Introduction

Students’ achievement motivation plays an essential role in everyday school life. The school environment often places emphasis on performance as students are confronted with different types of tasks and communicated expectations, by some of which they are also graded. As children and adolescents move through the educational system, emphasis on performance seems to increase (Anderman and Anderman, 1999; Eccles and Roeser, 2011) and, for example, in upper secondary education in Finland, performance is increasingly stressed as the success in studies has ramifications for forthcoming entry into further education. Accordingly, students’ achievements have an impact on their future, which is likely to cause study-related strain, but even so, students seem to cope with this challenge in different ways.

The goals students hold and adopt in achievement contexts is one likely factor contributing to this. Although the widely studied achievement goals have often been researched generally in relation to learning and studying (see Niemivirta et al., 2019), it is reasonable to assume that individuals also demonstrate variation in their preferences with respect to different subject domains; not all school subjects are alike (see Bong, 2004; Sparfeldt et al., 2015; Hornstra et al., 2016; Jansen in de Wal et al., 2016). Striving for a goal also requires investing time and effort, thus implying that there always is some subjective cost in play. This aspect of motivational trade-off is incorporated in the expectancy-value theory (Eccles et al., 1983) and, although empirically somewhat neglected until the recent years, has shown to provide insights particularly into the predictions of students’ avoidance motivation and behavior (e.g., avoidance goals, negative classroom affect: Jiang et al., 2018; drop-out intentions: Perez et al., 2014) and even well-being (Watt et al., 2019). Thus, a combined look at the perceived cost and achievement goals may help to explain students’ achievement behavior beyond positive purposes (Conley, 2012; Jiang et al., 2018) and, consequently, provide a more comprehensive view onto the complexity of students’ subject-specific motivational processes and their implications on study-related well-being.

Achievement motivation is a product of the interaction of the person and the context—including social environment and personal goals, beliefs, and emotions—and is, thus, also inseparably linked with an individual’s well-being. Achievement goals have indeed been found to have implications for different aspects of well-being, for example, the quality of engagement in schoolwork and emotional experiences in school (Daniels et al., 2008; Tuominen et al., 2020b). Building on this line of research, this study investigated (1) how different achievement goal orientations in relation to two key academic domains, mathematics and languages, in general upper secondary school combine to shape motivational profiles and (2) how those profiles predict student well-being both in terms of subject-specific perceived cost (i.e., how exhausting it is to study the subject, how much negative emotions are associated with it, and how much it requires giving up other valued alternatives) and more general academic well-being, such as school burnout (i.e., how exhausted, cynical, or inadequate a student feels in relation to school demands) and school engagement (i.e., how engaged a student is in schoolwork).

### Achievement Goal Orientations

According to the achievement goal theorists, the goals individuals are pursuing create a kind of framework within which the individuals interpret situations and which further produce patterns of cognition, emotion, and behavior (Dweck and Leggett, 1988; see also Senko et al., 2011). Basically, two lines of research differing in the level of specificity and operationalization exist: one that focuses on achievement goals, the specific end-states individuals strive for in a given context (Elliot, 1999), and another which looks at achievement goal orientations, the more general tendencies reflecting goal preferences and adoption (Nicholls, 1989; Dweck, 1992; for an overview, see Niemivirta et al., 2019). Here, we follow the latter line of research.

Studies on achievement goals differ also in which goals they take into account. Early research focused on two contrasting goals: mastery and performance (Dweck, 1986; Dweck and Leggett, 1988), that is, the goal of increasing competence and the goal of demonstrating competence. Both mastery and performance goals indicate an approach toward what the student considers as successful, but Nicholls et al. (1985) suggested that students’ classroom behavior can also be driven by avoidance (e.g., alienation or avoiding challenging tasks and putting in an effort), thus leading to the identification of (work-)avoidance goal (see also Dowson and McInerney, 2001).

A framework linking the definition of competence (intrapersonal *vs*. normative) with valence (approach *vs*. avoidance) took a different view on avoidance and suggested that both mastery and performance goals consist of two interdependent approach–avoidance components (e.g., seeking superiority *versus* avoiding inferiority in a performance goal; learning *versus* avoiding not learning in a mastery goal; 2 × 2 goal model; Elliot and McGregor, 2001). This division is currently widely accepted, although the mastery-avoidance dimension has still received less support. The prevalence and the relevance of mastery-avoidance goals among young students seems vague (Sideridis and Mouratidis, 2008; Bong, 2009), as it appears to be infrequent for school-aged children and adolescents, who are still improving their competence, to hold such goals (Lee and Bong, 2016).

Yet another refinement focuses, on one hand, on the different manifestations of mastery and, on the other hand, on the difference between absolute and relative success, leading to the specification of an outcome (Grant and Dweck, 2003) or mastery-extrinsic goals (Niemivirta, 2002). Seeking absolute success and good grades with no relative reference to how others succeed thus implicates the goal of mastery but, instead of alluding to the intrinsic process of learning, points to the extrinsic expressions of mastery, such as grades or teacher’s remarks. It could be said that this orientation signifies achievement, yet not competition (see also Brophy, 2005). A most recent, although yet less applied, classification of goals combines valence with another kind of definition of competence (i.e., in reference to task, self, or others) to create six distinct goals (Elliot et al., 2011). Lastly, some extensions incorporate social goals either as students’ social reasons for engaging in academic activities or as students’ strivings for social competence (e.g., Dowson and McInerney, 2001; Gonçalves et al., 2017; Litalien et al., 2017a).

Besides the differences in the classes of goals or goal orientations, studies have differed in whether they assess goals related to studying in general (i.e., items referring to general schoolwork) or regarding specific school subjects or domains (i.e., items referring to a certain subject, course, class, or task). What seems to be still lacking is an examination of the relative emphasis of students’ achievement goal orientations simultaneously in several subjects.

### Generality or Specificity in Achievement Goal Orientations Across Domains?

If a student is striving to outperform peers in mathematics, does that implicate that the same student will hold a similar performance goal in, for instance, English class? In other words, to what extent are achievement goal orientations domain-specific or general across subject domains? Students’ goals are shaped by both individual predispositions (e.g., beliefs about own abilities and intelligence; Dweck and Leggett, 1988) and situational factors (e.g., teacher and the close educational context or the larger cultural environment; Anderman and Anderman, 1999; Litalien et al., 2017b). These individual and situational factors contribute to the nature of achievement goal orientations in terms of their cross-domain generality *versus* specificity. For example, the theories of intelligence have been proposed to be domain-general rather than domain-specific (Dweck, 1986). Furthermore, regarding situational factors, domain-generality of goals can be expected among young children, as elementary school students mainly have their lessons in the same class and are taught by the same class teacher, and thus the classroom practices and evaluation criteria are likely rather similar even in different subjects (see Jansen in de Wal et al., 2016). Then again, some domain-specificity in goals could be assumed among older students, as they tend to endorse more differentiated conceptions of ability compared with young children and become more aware of their own interests and identities (Bong, 2001; Wigfield and Wagner, 2005) and their school environment includes more variation in terms of teachers, subjects, and classroom practices.

Indeed the domain-specificity of academic motivation has been shown to depend on students’ age so that high school students’ motivation is more differentiated compared to the motivation of younger middle school students (Bong, 2001; see also Hornstra et al., 2016). An examination of the associations of achievement goal orientations across subject domains might inform us about the extent to which they are a function of individual dispositions or situational influences (see Bong, 2004). Research shows certain achievement goal orientations to be more subject-specific and distinct, while others generalize more across multiple academic domains. Specifically, mastery orientation appears least generalizable, whereas performance-approach and performance-avoidance orientations demonstrate stronger correlations over subjects (Bong, 2001, 2004; Sparfeldt et al., 2015; Hornstra et al., 2016). Thus, mastery goals seem more context-dependent and connected to a specific task or content, while performance-tendencies appear more independent of the specific task at hand. Performance-related orientations’ strong between-domain relations might reflect the influence of more stable individual differences associated with ability concerns, such as the need for achievement, fear of failure, competitiveness, and susceptibility to normative concerns (Elliot and McGregor, 2001; see also Sparfeldt et al., 2015). Although cross-domain associations of work-avoidance goals have been studied less, evidence shows a strong correlation between avoidance goals in the domains of school and sport (Duda and Nicholls, 1992) and across six school subjects (i.e., mathematics, physics, chemistry, history, German, and English; Sparfeldt et al., 2015). Whether this reflects a more general attitude toward school or lack of interest or value in different subjects remains an open question.

To sum up, despite some subject-specific differentiation in achievement goal orientations in the course of students’ development, the between-domain correlations of goal orientations vary from moderate (mastery orientation) to high (performance- and avoidance-related orientations) among students of different ages (Bong, 2001, 2004; Sparfeldt et al., 2015; Hornstra et al., 2016). This, together with the finding showing goal orientations to be rather stable over time (Pulkka and Niemivirta, 2013; Lo et al., 2017; Tuominen et al., 2020b), implicates some degree of domain-generality; in other words, students seem likely to display certain goal preferences across contexts and subject domains despite some task- or situation-specific variation (see Niemivirta et al., 2019).

### Person-Oriented Approach and Achievement Goal Orientation Profiles

Achievement goals or goal orientations are not mutually exclusive despite being independent dimensions. Research has widely supported the notion that students can hold multiple goals simultaneously (e.g., Pintrich, 2000; Barron and Harackiewicz, 2001; Niemivirta, 2002; Daniels et al., 2008). Until now, many researchers have already explored the different combinations of achievement goals and their associations with diverse outcomes by utilizing a person-oriented approach (for reviews, see Wormington and Linnenbrink-Garcia, 2017; Niemivirta et al., 2019). The person-oriented approach (Bergman et al., 2003; Bergman and Trost, 2006) enables the extraction of groups of individuals according to the patterns they show in terms of the studied individual characteristics (e.g., achievement goal orientations) as well as examining how big a proportion of the sample shows a particular pattern and how specific patterns are related to the outcomes of interest. In this way, a person-oriented approach seems particularly well suited for examining a multiple-goal pursuit.

Within the numerous studies on achievement goal orientation profiles, there are variations in the achievement goal variables chosen for classification, sample characteristics, and analytical methods (see Niemivirta et al., 2019). Studies also differ in whether they examine domain-general or domain-specific achievement goal orientations. Reviews on these studies (Wormington and Linnenbrink-Garcia, 2017; Niemivirta et al., 2019) reveal that, despite these variations, there are notable similarities in the number and the nature of profiles identified. More specifically, the number of extracted profiles most commonly vary between three and six, and the profiles seem rather similar across studies, even regardless of the educational context. In prior research, the profiles studied have been most often domain-general (e.g., Pulkka and Niemivirta, 2013; Gonçalves et al., 2017; Tuominen et al., 2020b), and when examining subject-specific goal orientation profiles, they have mainly focused on a single domain, usually mathematics.

With respect to subject-specific profiles, the mathematics-related achievement goal profiles have usually included a predominantly mastery-oriented profile (e.g., primarily mastery-oriented, learning-oriented; Turner et al., 1998; Berger, 2012; Schwinger and Wild, 2012; Schwinger et al., 2016), a predominantly performance-oriented profile (e.g., moderately performance-oriented, low-mastery/high-performance; Pintrich, 2000; Schwinger et al., 2016), a high goal profile (e.g., high multiple goals, success-oriented; Luo et al., 2011; Jang and Liu, 2012; Schwinger and Wild, 2012; Schwinger et al., 2016), a combined mastery- and performance-approach yet low performance-avoidance goal profile (e.g., approach; Luo et al., 2011; see also Lo et al., 2017), a moderate goal profile (e.g., moderate multiple goals, moderate/indifferent, indifferent, diffuse; uncommitted; Turner et al., 1998; Luo et al., 2011; Schwinger and Wild, 2012; Schwinger et al., 2016; Lo et al., 2017), and a low goal profile (e.g., low multiple goals, amotivated; Jang and Liu, 2012; Schwinger et al., 2016), as well as a predominantly avoidance-oriented profile, in the few cases when avoidance goals have been included in the clustering (Berger, 2012). Goal profile studies focusing on other domains are scant. Regarding languages, Woodrow (2006) identified two profiles (i.e., adaptive and less adaptive learning) in English. There are some studies examining students’ goal profiles, for example, in science (Bae and Debusk-Lane, 2018), anatomy (Lee et al., 2017), and accounting (Dull et al., 2015), revealing mostly similar profiles as in mathematics.

To date, studies focusing on profiles in multiple subjects simultaneously are scarce. A study by Jansen in de Wal et al. (2016) is a rare example of such studies, as it explored achievement goal profiles in two domains—language and mathematics. They distinguished three different profiles in both domains among elementary school students: multiple goals, approach-oriented, and moderate/indifferent. Multiple goals profile was characterized by similar, rather high, scores on all achievement goals. Approach-oriented and indifferent profiles had average performance-approach and average to high mastery-approach goals, but the approach-oriented group also had lower performance-avoidance and higher mastery-approach goals than the indifferent group. In order to investigate the domain-specificity of the profiles, Jansen in de Wal et al. (2016) conducted latent profile analyses separately for the two subjects, classified cases into profiles based on their highest latent class probabilities, and compared those categorizations between language and mathematics. Profile membership was found to be relatively domain-general as 60% of the students had similar profiles in both domains. Still there was a significant number of students demonstrating different profiles for language and mathematics, thus showing also some domain-specificity in goal orientations (Jansen in de Wal et al., 2016).

### Achievement Goal Orientations, Perceived Cost, and Academic Well-Being

Some studies looking at students’ multiple goals have suggested that certain goal combinations might be associated with more adaptive patterns of coping and emotion than others (see Wormington and Linnenbrink-Garcia, 2017; Niemivirta et al., 2019). In the present study, we will address the adaptability of goal orientation profiles in terms of both subject-specific (i.e., cost) and more general (i.e., school engagement and burnout) aspects of student well-being.

Task values have been researched particularly in relation to different subjects as beliefs that influence students’ determination, performance, and choices (see, e.g., Wigfield et al., 2017). The expectancy-value theory (Eccles et al., 1983) includes four value components: intrinsic value, attainment value, utility value, and cost. The first three reflect the positive valence of a task, while cost refers to the perceived negative consequences and requirements of task engagement (Wigfield and Eccles, 1992; Barron and Hulleman, 2015; Gaspard et al., 2015). Earlier studies exploring cost included a single facet of opportunity cost (Conley, 2012; Trautwein et al., 2012) or used single factors which combined several facets together (Luttrell et al., 2010), while in later work, qualitatively different aspects of costs have been differentiated into separate subfacets, such as *effort required* (i.e., students’ perceptions of how much effort is required to succeed in a task or subject), *emotional cost* (i.e., the negative affective states a student may encounter in relation to different subjects and their demands), and *opportunity cost* (i.e., perceptions of whether engaging in the subject means having to give up on other valued activities) (e.g., Perez et al., 2014; Gaspard et al., 2015; see Wigfield et al., 2017; see also Flake et al., 2015, who differentiated a fourth subfacet: *outside effort cost*). These subfacets of cost are separate in meaning but often strongly associated (Flake et al., 2015; Gaspard et al., 2017, 2018).

Despite only few studies available, the more specific look at cost seems informative. Cost has shown to differentiate students’ adaptive and maladaptive motivational patterns (Conley, 2012), contribute to subsequent academic achievement (Conley, 2012; Hong et al., 2020), and predict retention intentions (Perez et al., 2014), and due to its association with debilitated well-being (i.e., depression, anxiety, and stress; Watt et al., 2019) and the adoption of avoidance motivation (Jiang et al., 2018), it also seems to play a role in student engagement. As to the associations between costs and achievement goals, the findings are scant and somewhat mixed due to variation in the assessment of the constructs (e.g., whether just one combined component or multiple subfacets of cost have been used). Nevertheless, cost has shown to be either unrelated or negatively correlated with mastery goals and positively with performance-approach, performance-avoidance, and work-avoidance goals (Conley, 2012; Jiang et al., 2018; Hong et al., 2020). Taken together, cost clearly plays a role in student motivation and engagement and seems particularly relevant in connection with students’ emotional experiences and well-being in school.

In addition to subject-specific cost, we also addressed more general student well-being, which can be examined in terms of either presence or absence of positive (e.g., engagement) and negative (e.g., burnout) indicators. School engagement is defined as a positive, fulfilling study-related state of mind characterized by energy, positive cognitive attitude toward studying, and being absorbed in studying (Salmela-Aro and Upadyaya, 2012). School burnout instead emerges as a negative response to a student’s continued difficulties in coping with school-related achievement pressures and is defined as exhaustion at school, cynicism toward school, and feelings of inadequacy (Salmela-Aro et al., 2009). Prior studies on the relations between achievement goal orientations, school engagement, and burnout (e.g., Tuominen-Soini et al., 2008, 2012; Tuominen et al., 2020b) have shown mastery-intrinsic orientation to be related to high engagement and low burnout (especially cynicism and inadequacy) and mastery-extrinsic orientation to be similarly associated with high engagement and low cynicism and inadequacy but, also, among secondary school students, positively associated with exhaustion. Furthermore, performance-approach orientation has been positively yet rather weakly correlated with engagement, exhaustion, and inadequacy, whereas performance-avoidance has been negatively yet weakly correlated with engagement and strongly positively correlated with all dimensions of burnout. Avoidance orientation has shown a strong negative correlation with engagement and strong positive correlations with cynicism and inadequacy. It seems relevant to consider students’ school engagement and burnout, as they are linked not only to achievement goal orientations (Tuominen-Soini et al., 2012) but also to various significant educational outcomes, such as educational aspirations and attainment, dropout, and academic achievement (Fredricks et al., 2004; Salmela-Aro and Upadyaya, 2012, 2017; Korhonen et al., 2014; Madigan and Curran, 2020; Widlund et al., 2020).

Research employing a person-oriented approach has consistently demonstrated the adaptability of mainly mastery and combined mastery and performance-approach goal profiles. For instance, mastery-oriented students have displayed a positive pattern of academic and emotional functioning, including high enjoyment, engagement, and academic achievement and, at the same time, low anxiety, negative affect, and perceived costs (Daniels et al., 2008; Luo et al., 2011; Schwinger et al., 2016; Gonçalves et al., 2017; Hong et al., 2020; Tuominen et al., 2020b). Students simultaneously pursuing both mastery and performance-approach goals (e.g., approach-oriented or success-oriented) have also exhibited high self-esteem, school value, and engagement (Tuominen-Soini et al., 2008, 2011, 2012) as well as high math-related self-efficacy, self-concept, metacognition, effort, and achievement (Luo et al., 2011; Jang and Liu, 2012; Lo et al., 2017). Interestingly, however, students holding multiple goals have also shown to express exhaustion at school, stress, fear of failure, and anxiety despite their often high enjoyment and engagement (Daniels et al., 2008; Tuominen-Soini et al., 2008, 2011; Jang and Liu, 2012; Zhang et al., 2016; Tuominen et al., 2020b).

Performance-approach goals, if coupled with high mastery goals, thus entail some favorable outcomes, but it seems clear that endorsing mainly performance goals and especially performance-avoidance goals leads to unfavorable concomitants. For example, the combination of high performance-avoidance goals and relatively low mastery goals has been linked with low effort and achievement, high anxiety and negative affect, and low self-esteem (Pintrich, 2000; Tapola and Niemivirta, 2008; Tuominen-Soini et al., 2008; Luo et al., 2011). Students exhibiting a moderate goal profile have shown to display moderate values also on various outcomes, such as engagement, school value, exhaustion, self-esteem, and math-related self-efficacy and self-concept (Turner et al., 1998; Tuominen-Soini et al., 2008; Schwinger et al., 2016; Lo et al., 2017; Tuominen et al., 2020b). In turn, students who exhibit low goal profiles have shown to manifest clearly less functional profile with regard to academic and socio-emotional functioning; for example, low performance, low enjoyment, high anxiety, and high boredom in mathematics (Conley, 2012; Jang and Liu, 2012). Finally, students mainly emphasizing work-avoidance orientation have shown to exhibit the most unfavorable outcomes, for instance, depressive symptoms, cynicism, low engagement and valuing of school, relatively poor achievement, and grade retention (Kolić-Vehovec et al., 2008; Tuominen-Soini et al., 2008, 2012; Peixoto et al., 2016) and, in mathematics, low individual interest, utility value, and perceived ability and high anxiety (Berger, 2012).

To summarize, students with high mastery or high approach goal (i.e., mastery- and performance-approach) profiles consistently show more adaptive patterns of academic and emotional functioning than students with predominantly performance-oriented, moderate goal, low goal, or work-avoidant profiles (Wormington and Linnenbrink-Garcia, 2017; Niemivirta et al., 2019).

### The Present Study

Previous research informs us about the cross-domain generality and specificity of achievement goal orientations (Bong, 2001, 2004; Sparfeldt et al., 2015; Hornstra et al., 2016), but less is known about how this translates into achievement goal orientation profiles. As such profiles have usually been researched in relation to studying in general, or in one subject only, studies examining students’ goal profiles in multiple subjects are lacking. The one study we are aware of compared achievement goal orientation profiles in language and mathematics and found some support for both generality across domains (i.e., 60% overlap) and domain-specificity, implicating the influence of more general personal tendencies on the profiles as well as some subject-specific preferences (Jansen in de Wal et al., 2016). Grounding on this, the first objective of the present study was to investigate what kinds of subject-specific achievement goal orientation profiles in mathematics and English can be identified among Finnish general upper secondary school students.

For this, we followed an operationalization of achievement goal orientations that represents a wide array of goals relevant in the classroom (i.e., mastery-intrinsic, mastery-extrinsic, performance-approach, performance-avoidance, and avoidance; Niemivirta, 2002; Niemivirta et al., 2019). This conceptualization grounds on the original division into mastery, performance, and work-avoidance goals (e.g., Nicholls, 1984; Nicholls et al., 1985; Dweck, 1986), with later distinctions into approach- and avoidance-components of performance goals (Elliot and Harackiewicz, 1996) and intrinsically and extrinsically based mastery goals (Niemivirta, 2002; Grant and Dweck, 2003). A person-oriented approach, by means of latent profile analysis (LPA), was adopted to capture multiple dimensions of achievement goal orientations in the two subjects as they co-occur in upper secondary studies.

Previous research also implies that the goals students seek to attain may contribute to their perceived investment in time and effort (e.g., Tuominen-Soini et al., 2008; Luo et al., 2011) and that this might vary depending on the school subject. This, in turn, might also become manifested in students’ study-related experiences (Daniels et al., 2008; Tuominen-Soini et al., 2012). Accordingly, our second aim was to investigate how the different achievement goal orientation profiles predict subject-specific perceived cost and academic well-being. In line with recent research demonstrating the added value of considering multiple dimensions of cost, we also focused on three different aspects of it: effort required, emotional cost, and opportunity cost (Perez et al., 2014; Gaspard et al., 2015). As to more general well-being, we included both positive and negative indicators since their relative presence or absence might have different implications. For this, we looked at students’ experiences of school engagement and school burnout, of which the latter further included three components: exhaustion, cynicism, and inadequacy (Salmela-Aro et al., 2009).

Based on earlier person-oriented studies (see Wormington and Linnenbrink-Garcia, 2017) and Niemivirta et al., 2019), we expected to identify several distinct achievement goal orientation profiles. Considering especially studies conducted in the domain of mathematics (e.g., Turner et al., 1998; Luo et al., 2011; Berger, 2012; Jang and Liu, 2012; Schwinger and Wild, 2012; Schwinger et al., 2016), we anticipated at least groups of students with predominantly mastery goal profile (e.g., mastery-oriented), a combined mastery and performance goal profile (e.g., success-oriented), a moderate goal profile (e.g., indifferent), and a mainly avoidance goal profile (e.g., avoidance-oriented). In addition, despite the limited prior evidence on the degree of cross-domain generality *versus* specificity in goal orientation profiles, but given the pair of school subjects in question (i.e., mathematics *versus* foreign language), we expected some profiles to demonstrate domain-specificity (see Jansen in de Wal et al., 2016).

Collating the previous research together and drawing particularly from Daniels et al. (2008), Tuominen-Soini et al. (2008, 2012), Luo et al. (2011), Lo et al. (2017), and Jiang et al. (2018) students with profiles high in mastery were anticipated to demonstrate adaptive well-being (e.g., low cost, high engagement, low burnout), students with profiles high in both mastery and performance were anticipated to demonstrate maladaptive along with adaptive well-being (e.g., high cost, high engagement, high exhaustion), students with profiles characterized by average scores on all orientations were anticipated to show moderate well-being (e.g., average cost, engagement, and burnout), and students with profiles high on avoidance goals were anticipated to demonstrate maladaptive academic well-being (e.g., low engagement, high cynicism), that is, as suggested by Watt et al. (2019), besides the consistently adaptive (e.g., mastery-oriented) and maladaptive (e.g., avoidance-oriented) profiles, we also expected to find asynchronous profiles (e.g., success-oriented), in which not only achievement goals but also costs would be high and which might further exert some deleterious effects on academic well-being (e.g., high emotional exhaustion and stress; see also Tuominen-Soini et al., 2008), regardless of high engagement (Tuominen et al., 2020b).

## Materials and Methods

### Participants and Procedure

Altogether 434 general upper secondary school students (*M*_age_ = 16.7, SD = 0.94; 179 girls, 255 boys) participated in the study by filling in online questionnaires. The participants were from all grades (53% first year, 25% second year, 19% third year, and 2% fourth or fifth year students) of one general upper secondary school in Southern Finland. In Finland, after the compulsory 9-year comprehensive school (children aged 7–16), students can opt either for general (academic track) or vocational (vocational track) upper secondary education. The general upper secondary school is based on courses with no specified year classes; in practice, students choose courses according to their individual study programs. The completion of general upper secondary school usually takes 3 years, but due to the course-based syllabus, it may also take less time or longer. Data collection took place mainly during regular classes, that is, only students who were absent during those classes filled in the questionnaires on their free time. Participation was voluntary, and the participants were assured of the confidentiality of their responses. The ethical review board in humanities and social and behavioral sciences (University of Helsinki) has reviewed the study and stated that it is ethically acceptable.

### Measures

#### Subject-Specific Achievement Goal Orientations

Students’ achievement goal orientations were assessed by using an instrument originally developed by Niemivirta (2002; Niemivirta et al., 2019) and validated in several previous studies (e.g., Tuominen-Soini et al., 2008, 2012; Pulkka and Niemivirta, 2013; Rawlings et al., 2017; Tuominen et al., 2020b). For the purposes of the present study, the instrument was modified to assess achievement goal orientations in mathematics and English. The instrument comprised 15 items measuring five separate achievement goal orientations (three items each) for both subjects: *mastery-intrinsic* (e.g., “An important goal for me in my studies is to learn as much as possible”), *mastery-extrinsic* (e.g., “It is important to me that I get good grades”), *performance-approach* (e.g., “An important goal for me in school is to do better than the other students”), *performance-avoidance* (e.g., “I try to avoid situations in which I may fail or make mistakes”), and *avoidance* (e.g., “I am particularly satisfied if I do not have to work much for my studies”) orientations. In the questionnaire, the item stems were presented on the left and the subjects (mathematics and English) on the right in separate columns, with Likert-type scales ranging from 1 (not at all true) to 7 (completely true).

Analyses concerning the structural validity of all scales were first conducted using confirmatory factor analysis (CFA) in M*plus* 8 (Muthén and Muthén, 2012–2017). Models were specified with items as indicators for their assigned latent constructs only. Maximum likelihood estimation was used to generate all solutions. The following indices were used to evaluate goodness of fit: comparative fit index (CFI ≥ 0.95; Bentler, 1990), root mean square error of approximation (RMSEA < 0.06; Steiger, 1990), and standardized root mean square residual (SRMR < 0.08; Hu and Bentler, 1998). According to Browne and Cudeck (1993), values between 0.05 and 0.08 for RMSEA suggest reasonable error of approximation, while RMSEA ≥ 0.10 suggests poor fit. The initial CFAs on achievement goal orientations (i.e., mastery-intrinsic, mastery-extrinsic, performance-approach, performance-avoidance, and avoidance) in mathematics and English described the data rather well, χ^2^(80) = 312.44/280.96, *p* < 0.001, CFI = 0.94/0.93, RMSEA = 0.082/0.076, SRMR = 0.064/0.060 (for mathematics and English, respectively). However, according to the modification indices, error covariances between one pair of similarly worded items were freed for both models (for factor loadings, residual variances, and other details, see Supplementary Table S1) and, consequently, the data showed an acceptable fit for both models, χ^2^(79) = 261.94/261.23, *p* < 0.001, CFI = 0.95/0.94, RMSEA = 0.073/0.073, SRMR = 0.060/0.060, thus verifying the hypothesized structures.

#### Subject-Specific Cost

Students’ perceived cost was assessed by utilizing a subscale of an instrument (Gaspard et al., 2015; see also Jiang, 2015) developed to capture the multidimensionality of value beliefs. Three dimensions of cost were assessed (three items each): *effort required* (e.g., “Dealing with math/English drains a lot of my energy”), *emotional cost* (e.g., “Doing math/English makes me really nervous”), and *opportunity cost* (e.g., “I have to give up a lot to do well in math/English”). The cost item stems were presented on the left and the subjects (mathematics and English) on the right in separate columns, with seven-point Likert-type scales ranging from 1 (not at all true) to 7 (completely true).

The initial CFAs on subject-specific cost (i.e., effort required, emotional cost, opportunity cost) described the data rather well, χ^2^(24) = 181.07/102.98, *p* < 0.001, CFI = 0.93/0.97, RMSEA = 0.123/0.087, SRMR = 0.056/0.032 (for mathematics and English, respectively), although the RMSEA values were elevated. Based on the modification indices, for mathematics, one item (“Learning mathematics exhausts me”) was excluded from further analysis and, for both mathematics and English, error covariances between one pair of similarly worded items were freed (see Supplementary Table S2). These modifications improved the fit of the models, χ^2^(16/23) = 56.00/64.48, *p* < 0.001, CFI = 0.98/0.98, RMSEA = 0.076/0.065, SRMR = 0.030/0.026.

#### School Engagement and School Burnout

Students’ engagement was measured by utilizing the schoolwork engagement inventory (EDA) by Salmela-Aro and Upadyaya (2012). It consists of nine items measuring a student’s energy (e.g., “At school I am bursting with energy”), dedication (e.g., “I find the schoolwork full of meaning and purpose”), and absorption (e.g., “Time flies when I am studying”) with respect to schoolwork. Here all nine items were deemed to indicate students’ overall school engagement. Responses were given on a Likert-type scale ranging from 0 (never) to 6 (everyday). School Burnout Inventory (Salmela-Aro et al., 2009) was used to assess three dimensions of school burnout: *exhaustion* (four items, e.g., “I feel overwhelmed by my schoolwork”), *cynicism* (three items, e.g., “I feel that I am losing interest in my schoolwork”), and *inadequacy* (three items, e.g., “I often have feelings of inadequacy in my schoolwork”). Students answered on a Likert-type scale from 1 (completely disagree) to 6 (completely agree).

Finally, a CFA for academic well-being measures (i.e., school engagement, exhaustion, cynicism, and inadequacy; see Supplementary Table S3) was conducted. The model fit the data rather well, χ^2^(146) = 442.50, *p* < 0.001, CFI = 0.93, RMSEA = 0.069, SRMR = 0.054, and the fit was further improved through correlating measurement errors between one pair of items measuring engagement, χ^2^(145) = 415.32, *p* < 0.001, CFI = 0.94, RMSEA = 0.066, SRMR = 0.053.

In summary, the confirmatory factor analyses showed that the hypothesized models fitted the data well. Descriptive statistics, internal consistencies (Cronbach’s alphas), and bivariate Pearson correlations for all variables are presented in Table 1.

**TABLE 1:** Bivariate correlations, descriptive statistics, and internal consistencies for all variables.

Measures		1.	2.	3.	4.	5.	6.	7.	8.	9.	10.	11.	12.	13.	14.	15.	16.	17.	18.	19.	20.
1.	MI_M_	–																			
2.	ME_M_	0.77**	–																		
3.	PAP_M_	0.47**	0.59**	–																	
4.	PAV_M_	0.18**	0.20**	0.43**	–																
5.	AV_M_	−0.27**	0.13**	0.09	0.16**	–															
6.	MI_E_	0.51**	0.36**	0.12*	0.09	–0.09	–														
7.	ME_E_	0.34**	0.44**	0.22**	0.06	0.05	0.66**	–													
8.	PAP_E_	0.12*	0.19**	0.63**	0.32**	0.21**	0.28**	0.51**	–												
9.	PAV_E_	0.08	0.10*	0.33**	0.79**	0.11*	0.08	0.11*	0.34**	–											
10.	AV_E_	–0.09	0.02	0.22**	0.14**	0.73**	−0.23**	–0.02	0.22**	0.15**	–										
11.	EFF_M_	–0.02	–0.06	0.12*	0.31**	0.11*	0.13**	0.08	0.19**	0.29**	–0.03	–									
12.	EMO_M_	−0.30**	−0.31**	–0.07	0.21**	0.31**	–0.00	–0.04	0.17**	0.23**	0.13**	0.67**	–								
13.	OPP_M_	0.02	–0.01	0.16**	0.35**	0.08	0.07	–0.04	0.21**	0.25**	0.01	0.62**	0.56**	–							
14.	EFF_E_	0.11*	0.08	0.15**	0.20**	–0.06	0.03	−0.18**	–0.05	0.22**	–0.04	0.42**	0.29**	0.33**	–						
15.	EMO_E_	0.03	–0.00	0.17**	0.25**	0.02	−0.15**	−0.32**	–0.05	0.24**	0.10*	0.29**	0.39**	0.34**	0.74**	–					
16.	OPP_E_	0.10*	0.06	0.22**	0.28**	–0.06	–0.03	−0.22**	0.04	0.25**	–0.03	0.34**	0.28**	0.59**	0.69**	0.69**	–				
17.	ENG	0.46**	0.39**	0.24**	0.10*	−0.31**	0.37**	0.26**	0.09	0.06	−0.26**	–0.01	−0.18**	0.03	0.09	–0.04	0.08	–			
18.	EXH	–0.01	0.00	0.20**	0.39**	0.01	0.07	0.02	0.19**	0.38**	–0.02	0.46**	0.38**	0.43**	0.29**	0.30**	0.35**	0.02	–		
19.	CYN	−0.36**	−0.27**	–0.01	0.19**	0.33**	−0.28**	−0.20**	0.09	0.19**	0.26**	0.26**	0.40**	0.26**	0.16**	0.25**	0.19**	−0.43**	0.44**	–	
20.	INA	−0.19**	−0.16**	0.08	0.31**	0.25**	−0.14**	−0.10*	0.14**	0.33**	0.17**	0.38**	0.44**	0.38**	0.26**	0.28**	0.26**	−0.27**	0.58**	0.69**	–
	*M*	4.89	5.20	4.09	4.19	4.85	5.50	5.66	4.47	4.35	4.67	4.31	3.80	3.52	3.23	2.82	2.86	3.23	3.17	2.79	3.12
	SD	1.47	1.43	1.45	1.55	1.34	1.26	1.20	1.48	1.58	1.35	1.64	1.62	1.58	1.51	1.42	1.47	1.34	1.08	1.22	1.05
	α	0.90	0.91	0.74	0.82	0.72	0.87	0.85	0.73	0.81	0.71	0.77	0.84	0.81	0.87	0.79	0.82	0.92	0.78	0.83	0.71

*p < 0.05, **p < 0.01. M, mathematics; E, English; MI, mastery-intrinsic orientation; ME, mastery-extrinsic orientation; PAP, performance-approach orientation; PAV, performance-avoidance orientation; AV, avoidance orientation; EFF, effort required; EMO, emotional cost; OPP, opportunity cost; ENG, school engagement; EXH, exhaustion; CYN, cynicism; INA, inadequacy; α, Cronbach’s alpha.

#### Correlations Between Variables

The correlational results between achievement goal orientations within a subject, between subjects, in relation to cost and well-being as well as between cost, engagement, and burnout showed meaningful and expected relations (see Table 1). Most importantly, although the between-domain (i.e., mathematics and English) correlations were all rather high, the correlations across the two subjects were higher for performance-approach (*r* = 0.63), performance-avoidance (*r* = 0.79), and avoidance (*r* = 0.73) orientations than for mastery-intrinsic (*r* = 0.51) and mastery-extrinsic (*r* = 0.44) orientations, which is in line with prior research (Bong, 2001, 2004; Sparfeldt et al., 2015; Hornstra et al., 2016). There were some interesting differences in the associations between goal orientations and the different subfacets of cost. For example, in math, mastery-intrinsic and mastery-extrinsic orientations were negatively correlated with emotional cost but unrelated with effort required and opportunity cost, while performance-approach orientation was positively correlated with effort required and opportunity cost but unrelated with emotional cost. Performance-avoidance orientation was positively related with all dimensions of cost, whereas avoidance orientation had the strongest positive correlation with emotional cost. In English, the associations were otherwise similar, but mastery-extrinsic orientation was negatively correlated with all dimensions of cost and performance-approach orientation was unrelated with cost. Regarding academic well-being, engagement was correlated positively with mastery-related orientations and negatively with avoidance orientation. Engagement was positively associated with performance-approach orientation only in math. Exhaustion correlated positively with performance-related orientations in both subjects. Cynicism and inadequacy were associated negatively with mastery-related orientations and positively with performance-avoidance and avoidance orientations in both subjects. All subfacets of cost were positively linked with all dimensions of burnout.

### Data Analyses

#### Latent Profile Analysis

The first main aim was to investigate what kinds of subject-specific achievement goal orientation profiles can be extracted. Thus, students with similar patterns of achievement goal orientations were identified through a probabilistic model-based variant of cluster analysis, the LPA (Vermunt and Magidson, 2002), using M*plus* 8. The LPA was used to identify the smallest number of latent classes (groups) that adequately describes the associations among observed continuous variables of five achievement goal orientations in two different subjects (i.e., 10 clustering variables). Classes are added stepwise until the model optimally fits the data. Akaike Information Criterion (AIC), Bayesian Information Criterion (BIC), sample-size adjusted BIC (SABIC), and Vuong–Lo–Mendell–Rubin (VLMR), and adjusted Lo–Mendell–Rubin (LMR) likelihood ratio tests were used as statistical criteria for determining the optimal number of profiles. The model with lower AIC, BIC, and SABIC values is considered to provide a better fit to the data, and *p*-values smaller than 0.05 for VLMR and LMR suggest that the model with one less class should be rejected in favor of the estimated model (Lo et al., 2001). In addition, when comparing different models, classification quality (entropy value > 0.70), meaningfulness, and interpretableness of the latent classes as well as conformity of the solutions with relation to theory and previous research were taken into consideration (Marsh et al., 2009).

#### The BCH Method

The second aim was to explore how students with diverse goal orientation profiles differ with respect to subject-specific cost and academic well-being. After the final profile solution was determined, the BCH method (Bolck–Croon–Hagenaars approach; Bolck et al., 2004) in M*plus* (see Asparouhov and Muthén, 2020) was applied to examine how the continuous distal outcomes differ as a function of the goal orientation profiles. The BCH method involves performing a weighted ANOVA, with weights that are inversely related to the classification error probabilities (Bakk et al., 2013; Bakk and Vermunt, 2016). It provides the results of equality tests that compare class-specific means of the distal outcomes across latent profiles (Asparouhov and Muthén, 2020). The BCH method avoids shifts in latent classes and has been demonstrated in simulation studies to be a robust approach (Bakk and Vermunt, 2016). Furthermore, to check how gender predicts profile membership, gender was added as a covariate to the model using the auxiliary R3STEP command in M*plus* (Asparouhov and Muthén, 2014), producing output to be interpreted as multinomial logistic regression.

## Results

### Subject-Specific Achievement Goal Orientation Profiles

**FIGURE 1:**
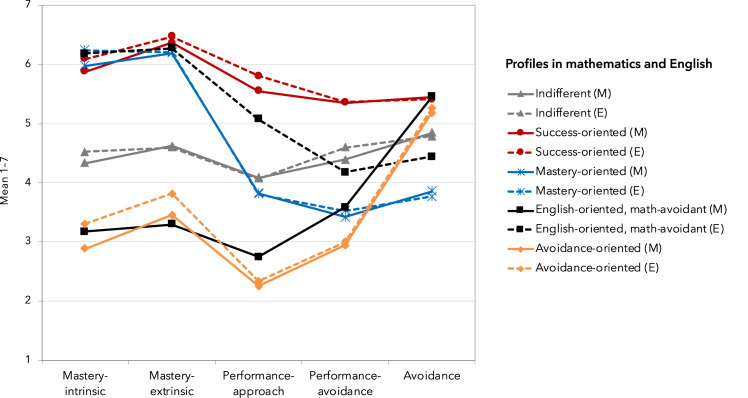
Achievement goal orientation profiles of the five groups in mathematics and English. Latent profile analysis was based on 10 clustering variables, that is, five achievement goal orientations in two subjects. The solid lines represent profiles in mathematics (M), and the broken lines represent profiles in English (E).

The first main goal of this study was to examine the kinds of goal orientation profiles that can be found among general upper secondary school students simultaneously in mathematics and English. The results from a series of LPAs (Table 2) showed that AIC, BIC, and SABIC decreased when additional latent classes were added, up to the six-class solution. However, after the four- and five-class solutions, the decrease stabilized. The *p*VLMR, *p*LMR, and high entropy provided support for the five-class solution. Along with the entropy value of 0.88, the average latent profile probabilities for the most likely latent profile assignment (0.95, 0.92, 0.93, 0.91, and 0.93, respectively; Table 3) pointed to a clear classification obtained with this solution. Furthermore, the group sizes were reasonable, and profiles were both qualitatively informative as well as compatible with previous research and theory. Thus, five distinct groups were identified, representing different goal orientation combinations in the two subjects, and they were labeled according to the score mean profiles as (1) *indifferent*, (2) *success-oriented*, (3) *mastery-oriented*, (4) *English-oriented*, *math-avoidant*, and (5) *avoidance-oriented* (see Figure 1 for the mean score profiles of the five groups in the two subjects and Table 4 for pairwise comparisons on mean values based on the BCH method).

**TABLE 2:** Fit indices for latent profile analyses.

*k*	AIC	BIC	SABIC	*p*_*VLMR*_	*p*_*LMR*_	Entropy	Group sizes
1	15,242.607	15,324.068	15,260.599	–	–	–	434
2	14,584.561	14,710.825	14,612.448	0.0000	0.0000	0.841	204, 230
3	14,286.987	14,458.055	14,324.770	0.1278	0.1309	0.835	111, 197, 126
4	14,000.328	14,216.200	14,048.007	0.0131	0.0140	0.864	61, 132, 116, 125
5	13,830.896	14,091.571	13,888.469	0.0375	0.0391	0.884	27, 108, 125, 62, 112
6	13,695.104	14,000.583	13,762.573	0.1129	0.1164	0.884	66, 25, 60, 120, 109, 54

*k, number of latent profiles in the model; AIC, Akaike Information Criterion; BIC, Bayesian Information Criterion; SABIC, sample-size adjusted BIC; p_VLMR_, Vuong–Lo–Mendell–Rubin likelihood ratio test; p_LMR_, Lo–Mendell–Rubin adjusted likelihood ratio test.*

**TABLE 3:** Average latent profile probabilities for most likely profile membership (row) by latent profile (column).

Profile	1	2	3	4	5
1. Avoidance-oriented	*0.954*	0.000	0.042	0.004	0.000
2. Mastery-oriented	0.000	*0.916*	0.031	0.007	0.045
3. Indifferent	0.016	0.026	*0.927*	0.013	0.017
4. English-oriented, math-avoidant	0.004	0.020	*0.057*	*0.908*	0.010
5. Success-oriented	0.000	0.046	0.018	0.004	*0.932*

*Values in italics represent the average posterior probability associated with the profiles to which students were assigned.*

**TABLE 4:** Mean differences in achievement goal orientations between the latent goal orientation profiles based on the Bolck–Croon–Hagenaars (BCH) method.

	Indifferent		Success-oriented		Mastery-oriented		English-oriented, math-avoidant		Avoidance-oriented			
Variable	*M*	*SE*	*M*	*SE*	*M*	*SE*	*M*	*SE*	*M*	*SE*	χ*^2^*	*p*
Mastery-intrinsic_M_	4.29	0.08	5.93_b_	0.09	6.08_b_	0.09	3.05_a_	0.15	2.78_a_	0.26	599.986	<0.001
Mastery-extrinsic_M_	4.59	0.08	6.43_b_	0.07	6.28_b_	0.07	3.11_a_	0.15	3.37_a_	0.18	877.941	<0.001
Performance-approach_M_	4.11	0.09	5.69	0.09	3.74	0.14	2.64	0.14	2.11	0.20	529.664	<0.001
Performance-avoidance_M_	4.48	0.11	5.48	0.13	3.27_a_	0.15	3.46_a_	0.21	2.86_a_	0.25	168.472	<0.001
Avoidance_M_	4.81_a_	0.11	5.57_b_	0.12	3.68	0.12	5.55_b_	0.17	5.28_ab_	0.29	139.364	<0.001
Mastery-intrinsic_E_	4.41	0.09	6.11_a_	0.09	6.30_a_	0.07	6.31_a_	0.11	3.18	0.23	488.832	<0.001
Mastery-extrinsic_E_	4.47	0.08	6.54_b_	0.07	6.24_a_	0.08	6.37_ab_	0.10	3.76	0.23	546.575	<0.001
Performance-approach_E_	4.04	0.10	5.98	0.09	3.63	0.14	5.18	0.17	2.20	0.22	436.507	<0.001
Performance-avoidance_E_	4.65	0.11	5.50	0.15	3.36_a_	0.15	4.11	0.24	2.90_a_	0.27	130.900	<0.001
Avoidance_E_	4.78_ab_	0.10	5.53_c_	0.13	3.62	0.13	4.43_a_	0.18	5.34_bc_	0.26	112.099	<0.001

*Means within a row sharing the same subscripts are not significantly different at p < 0.05 level based on Wald test in the BCH method. M, mathematics; E, English.*

*Indifferent* students (29%) had average scores on all orientations, that is, they displayed a moderate goal profile with no peak on any orientation. *Success-oriented* students (26%) demonstrated high multiple goals in mathematics and English, as they expressed a strive for both absolute and relative success as well as for learning and gaining competence in both domains. Interestingly, they scored rather high also on avoidance orientation in both subjects. *Mastery-oriented* students (25%) emphasized mastery-intrinsic and mastery-extrinsic orientations in both mathematics and English and, in turn, scored relatively low on performance-related orientations and the lowest on avoidance. *English-oriented*, *math-avoidant* students (14%) showed the most distinct domain-specificity in the achievement goal orientations out of all the groups. They aimed for learning, getting good grades, and outperforming others in English but had very low aspirations toward learning and succeeding in mathematics. They were also characterized by high avoidance orientation in mathematics. In turn, the small group of *avoidance-oriented* students (6%) scored high on avoidance in both domains and, at the same time, low on all mastery- and performance-related orientations.

Tests of multinominal logistic regressions indicated that girls were significantly more likely than boys to be members of the mastery-oriented profile compared to the success-oriented profile (–0.676, *p* < 0.05) and also to be members of the English-oriented, math-avoidant profile compared to the success-oriented (−0.967, *p* < 0.05) and indifferent (−0.822, *p* < 0.05) profiles.

### Profile Differences in Subject-Specific Cost and Academic Well-Being

In order to further illustrate the characteristics of the motivational profiles, we investigated how students with different subject-specific goal orientation profiles differed in terms of subject-specific perceived cost (i.e., effort required, emotional cost, opportunity cost) and academic well-being (i.e., school engagement, school burnout) by employing the BCH method.

The results of the equality tests of means across profiles using the BCH procedure showed that the groups differ significantly in effort required, emotional cost, and opportunity cost in mathematics and English (Table 5). For example, the success-oriented students reported higher effort required related to studying math than the mastery-oriented, avoidance-oriented and indifferent students. The English-oriented, math-avoidant students also scored relatively high in effort required. Regarding emotional cost related to studying math, students in the English-oriented, math-avoidant group scored the highest, followed by the indifferent, success-oriented, and avoidance-oriented students, who did not differ significantly from each other. Mastery-oriented students, in turn, perceived studying math as clearly less emotionally burdening compared to all other students. Opportunity cost in mathematics was relatively high among the success-oriented and indifferent students and low among the avoidance- and mastery-oriented students. Overall, for each group, the perceived cost seemed to be higher in mathematics than in English. As could be expected, students in the English-oriented, math-avoidant group scored lower in effort required and emotional cost in English compared to the indifferent and success-oriented students. Concerning opportunity cost in English, the indifferent and success-oriented students scored higher than the mastery-oriented, avoidance-oriented, and English-oriented, math-avoidant students.

**TABLE 5:** Mean differences in subject-specific cost and academic well-being between the latent goal orientation profiles based on the Bolck–Croon–Hagenaars (BCH) method.

	Indifferent		Success-oriented		Mastery-oriented		English-oriented, math-avoidant		Avoidance-oriented			
Variable	*M*	SE	*M*	SE	*M*	SE	*M*	SE	*M*	SE	χ^2^	*p*
Cost: effort_M_	4.34_bc_	0.14	4.75_d_	0.16	3.81_a_	0.17	4.62_cd_	0.28	3.64_ab_	0.37	19.508	0.001
Cost: emotional_M_	4.05_a_	0.13	4.02_a_	0.16	2.70	0.15	4.91	0.25	3.81_a_	0.32	77.031	<0.001
Cost: opportunity_M_	3.85_bc_	0.12	4.06_c_	0.17	2.88_a_	0.16	3.37_ab_	0.23	2.65_a_	0.33	37.493	<0.001
Cost: effort_E_	3.71_d_	0.12	3.32_cd_	0.18	3.09_bc_	0.16	2.56_a_	0.18	2.66_ab_	0.25	33.327	<0.001
Cost: emotional_E_	3.59	0.12	2.93_b_	0.16	2.29_a_	0.14	2.05_a_	0.17	2.57_ab_	0.22	74.801	<0.001
Cost: opportunity_E_	3.54_c_	0.12	3.16_c_	0.17	2.46_b_	0.15	2.05_ab_	0.16	1.89_a_	0.17	90.835	<0.001
School engagement	2.85_a_	0.12	3.64_b_	0.13	3.86_b_	0.13	2.74_a_	0.17	1.92	0.25	85.663	<0.001
Burnout: exhaustion	3.34_b_	0.10	3.51_b_	0.12	2.76_a_	0.10	3.20_b_	0.14	2.52_a_	0.21	34.634	<0.001
Burnout: cynicism	3.32_b_	0.11	2.86_a_	0.13	1.88	0.10	2.97_ab_	0.14	3.27_ab_	0.26	107.846	<0.001
Burnout: inadequacy	3.48_a_	0.09	3.34_a_	0.11	2.42	0.10	3.26_a_	0.12	3.04_a_	0.24	62.718	<0.001

*Means within a row sharing the same subscripts are not significantly different at p < 0.05 level based on Wald test in the BCH method. M, mathematics; E, English.*

Next, we proceeded to examine whether students with different subject-specific goal orientation profiles differ also with respect to well-being related to studying in general. The results demonstrated that the goal orientation groups differed significantly in school engagement and all dimensions of school burnout (Table 5). The mastery- and success-oriented students displayed the highest engagement, while the avoidance-oriented students scored clearly the lowest. For exhaustion, the success-oriented, indifferent, and English-oriented, math-avoidant students scored higher than the mastery- and avoidance-oriented students. The mastery-oriented students exhibited clearly the lowest cynicism and inadequacy in comparison to all other students.

## Discussion

Despite the abundance of studies on goal profiles, studies examining students’ achievement goal orientation profiles in multiple subjects are lacking, and therefore more information on the cross-domain generality *versus* specificity of profiles is needed. As striving for achievement-related goals involve investment in time and effort and since the implications of this might vary depending on the types of goals pursued, it is also of importance to examine how the different goal profiles contribute to students’ well-being. Accordingly, the present study aimed at investigating (1) what kinds of achievement goal orientation profiles in two key subjects, mathematics and English, can be identified among general upper secondary school students and (2) how students with different goal orientation profiles differ with regard to subject-specific cost and academic well-being.

### Achievement Goal Orientation Profiles: More Cross-Domain Generality Than Specificity

In identifying students’ subject-specific achievement goal orientation profiles, our analyses revealed, mainly in line with prior studies examining goal orientation profiles in relation to studying in general (e.g., Turner et al., 1998; Tuominen et al., 2020b) or in mathematics (e.g., Luo et al., 2011; Jang and Liu, 2012; Schwinger and Wild, 2012), five divergent profiles: (1) moderate multiple goals profile (*indifferent*), (2) high multiple goals profile (*success-oriented*), (3) predominantly mastery goal profile (*mastery-oriented*), (4) a profile reflecting positive motivation in English but high avoidance goals in mathematics (*English-oriented*, *math-avoidant*), and (5) predominantly avoidance goal profile (*avoidance-oriented*).

The *indifferent* students (29%) display a rather balanced and average endorsement of all goals in both subjects, that is, they do not demonstrate a tendency to favor any specific orientation. This finding concurs with previous person-oriented studies extracting a group of students displaying a moderate goal profile (e.g., Turner et al., 1998; Luo et al., 2011; Schwinger and Wild, 2012; Tuominen-Soini et al., 2012; Pulkka and Niemivirta, 2013; Jansen in de Wal et al., 2016; Schwinger et al., 2016; Lo et al., 2017). Also in line with prior research, the indifferent students form the largest group, suggesting that these students represent somewhat an average student who comprehends the importance of learning, gaining competence, and grades but is still rather undevoted to the realization of these goals (Tuominen-Soini et al., 2008, 2011; Lo et al., 2017). This slight reluctance to put in effort seems to apply across, and possibly beyond, the two domains.

The *success-oriented* students (26%) clearly value learning and developing competence but are also likely to place value on relative ability and avoiding demonstrating incompetence. This kind of group of students seemingly striving for multiple goals is often found (Luo et al., 2011; Jang and Liu, 2012; Schwinger and Wild, 2012; Pulkka and Niemivirta, 2013; Schwinger et al., 2016; Tuominen et al., 2020b). Interestingly, in this study, the success-oriented students endorsed both high performance-approach and performance-avoidance goals along with high mastery goals and even surprisingly high (work-)avoidance goals; hence, this profile denotes a pattern of combined approach and avoidance goals (see also Luo et al., 2011). This might seem logical when taking into consideration their wish to succeed, develop, and demonstrate competence in multiple subjects because avoiding making an effort when possible may be the only way to manage own limited resources. A recent study (Mouratidis et al., 2018) also found that performance-avoidance goals are negatively associated with challenge-seeking. These students, thus, strive for learning and succeeding but may not enjoy challenge as much as their mastery-oriented peers and are more prone to avoiding investing their time and effort. Thus, there clearly are some costs to endorsing performance- and avoidance-related goals. It should be mentioned that although person-oriented studies have also often extracted an approach profile (i.e., high mastery and performance-approach yet low performance-avoidance goals; e.g., Luo et al., 2011; Schwinger et al., 2016; Lo et al., 2017) and seldom also a purely performance-oriented profile (i.e., low mastery/high performance goals; see Wormington and Linnenbrink-Garcia, 2017), these profiles were not found here. Perhaps the performance-focused climate of the general upper secondary school (i.e., academic track) induces the co-occurring pursuits of superiority and avoiding inferiority.

The *mastery-oriented* students (25%) emphasize learning and developing competence (i.e., mastery-intrinsic orientation) as well as succeeding on extrinsic criteria (i.e., mastery-extrinsic orientation; focusing on absolute success without any reference to others) in both mathematics and English. In turn, they do not exhibit performance- or avoidance-focused tendencies. A group of students holding a dominant tendency toward learning is identified in the vast majority of goal profile studies, both in relation to mathematics (e.g., Turner et al., 1998; Berger, 2012; Schwinger and Wild, 2012; Schwinger et al., 2016) and studying in general (Kolić-Vehovec et al., 2008; Tapola and Niemivirta, 2008; Pulkka and Niemivirta, 2013; Peixoto et al., 2016; Zhang et al., 2016). Given that the main schoolwork focus for these students is on learning, understanding, improvement, and self-comparison, their motivational profile appears very favorable.

In addition to the four groups that were anticipated based on prior research, a novel group of *English-oriented*, *math-avoidant* students (14%) was identified. These students show the strongest domain-specificity in the achievement goal orientations, that is, they demonstrate distinct subject-specific preferences for goals related to learning and succeeding in mathematics and English. These students aim for learning, getting good grades, and outperforming others in English but have very low aspirations toward learning and succeeding in mathematics and also express elevated avoidance orientation in mathematics. Thus, they seem to have an approach goal profile in English (high mastery, high performance-approach, low performance-avoidance), yet in mathematics, they have a nearly equivalent profile to those of avoidance-oriented students (see Figure 1). The emergence of this group may reflect the affective differences related to the subjects in question. Research has often found high emotional stress and anxiety related to mathematics (e.g., Meece et al., 1990; Skaalvik, 2018), but studying English might not be as anxiety-provoking. Girls are more likely to belong to the English-oriented, math-avoidant profile than to the success-oriented or indifferent profiles, which might represent a susceptibility to stereotypical assumptions on the nature of mathematics and mathematical fields.

Finally, *avoidance-oriented* students (6%) are characterized by a lack of thrive to learn, succeed, or outperform others but rather a strong focus on avoiding schoolwork and minimizing effort in both mathematics and English. In this study, avoidance goals seemed rather domain-general as four groups out of five showed very similar levels in avoidance tendencies in both subjects, and only the English-oriented, math-avoidant students exhibited clearly different levels of avoidance in the two subjects. Thus, the students’ tendency to invest as little effort as possible in studies might be rather context-independent (see also Sparfeldt et al., 2015) and reflect a more general attitude toward school. Avoidance orientation is less frequently assessed in person-oriented studies, but when it has been included, studies have identified a small group of students who do not manifest focus on learning and performance typical among most students in the school context (Brdar et al., 2006; Kolić-Vehovec et al., 2008; Tuominen-Soini et al., 2008, 2011, 2012; Berger, 2012; Pulkka and Niemivirta, 2013). It seems important to include avoidance orientation in the assessment of students’ goals as it helps to grasp students’ manifold motivational aspirations in achievement contexts and facilitates recognizing a group of students exhibiting a particularly unfavorable, amotivated profile.

The issue of domain-specificity of achievement goal orientation profiles has been underemphasized in prior studies. The present study is one of the few investigating students’ goal orientation profiles in more than one subject simultaneously. Interestingly, when looking at the score mean profiles (see Figure 1), it seems evident that, overall, the profiles educed indicate more domain-generality than domain-specificity. Only one group—the English-oriented, math-avoidant—demonstrated a clear distinction between motivation in the two subjects. The only previous study, to our knowledge, examining domain-specificity of goal profiles (Jansen in de Wal et al., 2016) applied a slightly different analytical approach as they conducted LPAs for the achievement goal orientations (i.e., mastery-approach, performance-approach, performance-avoidance) separately in the two subjects, classified cases into profiles on the basis of their highest latent class probabilities, and, after that, compared the classifications between subjects (language and mathematics). Here the clustering of students was conducted for the achievement goal orientations (i.e., mastery-intrinsic, mastery-extrinsic, performance-approach, performance-avoidance, and avoidance) simultaneously in the two subjects in order to better capture students’ multiple goals and their emphases in different domains and to also make the interpretation of the subject-specific profiles as straightforward as possible. By employing this kind of an analytical approach, we were able to identify the novel goal orientation group of English-oriented, math-avoidant students.

In line with prior studies (Bong, 2001; Sparfeldt et al., 2015; Hornstra et al., 2016), goals linked to learning and developing competence were more context-dependent than other goals, but all cross-domain correlations were still rather high. The detected high cross-domain associations, in addition to the findings that achievement goal orientations and goal orientation profiles seem relatively stable even over time (Tuominen-Soini et al., 2011, 2012; Pulkka and Niemivirta, 2013; Lo et al., 2017; Tuominen et al., 2020b) and that the possible developments in achievement goals in one domain are likely to coincide with similar developments in another domain (i.e., language and math; Hornstra et al., 2016), lend support for the adopted view of goal orientations as a disposition reflecting students’ generalized tendencies to perceive and approach various achievement and learning settings in certain ways, that is, regardless of some fluctuation, many students are still inclined to hold certain goal preferences even across contexts (e.g., different subjects or domains) and over time (see also Niemivirta et al., 2019). Furthermore, when looking at the subject-specific goal orientation profiles, the findings of the present study as well as those of Jansen in de Wal et al. (2016) also support this conception by showing that the majority of students demonstrate rather domain-general achievement goal orientations, while a smaller proportion of students display more distinct differences between subjects. As Jansen in de Wal et al. (2016) concluded, it seems that students’ achievement goal orientations are formed by a combination of personal tendencies toward particular goals and different domain-specific components, such as students’ interest or perceived competence in the domain.

### Subject-Specific Achievement Goal Orientation Profiles Associated With Cost and Academic Well-Being

Earlier studies have addressed, for example, how domain-general achievement goal orientation profiles relate to domain-general school value, engagement, and affect (e.g., Tuominen-Soini et al., 2008, 2012; Gonçalves et al., 2017) and how math-specific goal profiles are associated with math-specific self-efficacy, self-concept, and anxiety (Luo et al., 2011; Jang and Liu, 2012; Lo et al., 2017). However, we know less about how subject-specific goal orientation profiles in several subjects are linked with both subject-specific perceived costs and more general study-related well-being. The examination of both goals and costs might be especially useful in revealing divergent patterns of subject-specific motivations and pressures (see Conley, 2012; Hong et al., 2020).

The findings of this study regarding the emotional outcomes of goal orientation profiles are in line with previous research demonstrating the adaptiveness of the high mastery and the combined mastery and performance goal profiles (for reviews, see Wormington and Linnenbrink-Garcia, 2017; Niemivirta et al., 2019). The mastery-oriented students demonstrate relatively low overall subject-specific cost, and especially the emotional costs (i.e., the negative affective states, such as feeling nervous, annoyed, or worried) related to studying both mathematics and English are low compared to other students. The mastery-oriented students are also highly engaged in school and report low levels of burnout, especially cynical attitude toward school (see also Meece and Holt, 1993; Turner et al., 1998; Tuominen-Soini et al., 2012; Tuominen et al., 2020b). Thus, the mastery-oriented students seem to show the most adaptive pattern of learning and academic well-being.

Success-oriented students clearly hold multiple goals in several subjects and, like mastery-oriented students, they are highly engaged in school (see also Luo et al., 2011; Tuominen et al., 2020b). Interestingly, such high strivings entail more or less emotional distress and strain (see Tuominen-Soini et al., 2008, 2012). In other words, even though focusing on mastery and performance simultaneously may result in positive outcomes as regards engagement, it might still come at a cost; the success-oriented students report relatively high levels of all subfacets of cost in both subjects, and they are also somewhat emotionally exhausted. Feeling fatigued seems probable due to their aims and efforts to learn, succeed, and outperform others combined with their high perceived costs, such as feelings that studying drains a lot of energy and requires giving up other valued alternatives. As has been noted in previous research, students aiming to succeed well often do but are also susceptible for negative effects on well-being (e.g., Tuominen-Soini et al., 2011, 2012). These findings reflecting the differences between mastery- and success-oriented students echo the results of Watt et al. (2019), who identified Positively engaged (i.e., high on expectancy-value constructs, low on costs) and Struggling ambitious (i.e., high on positive values and costs) students, who differed in that the Struggling ambitious students perceived high costs, accompanied with debilitated psychological well-being, but still no impairment in achievement striving.

As expected, students with moderate and avoidance-oriented profiles exhibit less adaptive profiles in terms of study-related well-being compared to the mainly mastery or combined mastery and performance goal profiles. The unwillingness of indifferent students to invest in the attainment of adaptive achievement goals coincides with relatively high perceived costs in both domains. Similarly, Hong et al. (2020) identified a Moderately Motivated group, which represented students with somewhat elevated tendency for social comparison as well as feeling that task engagement requires giving up something. The indifferent students also struggle to find meaning in their schoolwork as they express not only especially high cynicism but also rather high overall burnout (as high as the success-oriented students in all dimensions of burnout) and also moderate engagement in schoolwork. The passivity and the lack of engagement of indifferent students have been detected in earlier work (e.g., Turner et al., 1998; Tuominen et al., 2020b), yet also pointing out that these students do not undergo serious psychological distress. These findings support the suggestion of the indifferent student to represent a typical Finnish upper secondary school student, who understands the prevailing nature of upper secondary school and acknowledges the importance of learning and performing well but does not thrive to succeed.

Among the avoidance-oriented students, who aim to minimize effort in school, only emotional cost in mathematics is slightly elevated, but other subfacets of cost are low in both subjects. It seems natural that a student does not perceive studying as draining a lot of energy or as forcing oneself to give up other activities, if the goal is to get away with as little amount of work as possible. In line with previous research (e.g., Tuominen-Soini et al., 2012), this group demonstrates the lowest engagement out of all the groups and is characterized by relatively high cynical attitude toward school. The low level of achievement-related goals may reflect lack of perceived meaning in school which, in turn, could be demonstrated as cynicism. Note, however, that although this group expresses a rather maladaptive pattern of motivation and well-being, aiming to avoid schoolwork and not having concerns over succeeding is also associated with low exhaustion at school (as low as those of mastery-oriented students) and relatively low costs.

Finally, the perceived energy drainage and negative affective states (effort required and emotional cost) in studying mathematics seem particularly high among the English-oriented, math-avoidant students. With respect to studying English, however, these students display an adaptive motivational pattern with relatively high mastery and performance-approach goals and low costs. The results resemble those of Gaspard et al. (2018) in suggesting cost, and especially effort and emotional cost, to be subject-specific; this group expressed the strongest subject-specificity in both goal orientations and effort and emotional cost. This group is characterized by mixed goals in different domains, which is also reflected in their academic well-being; these students display rather average engagement but also slightly elevated burnout.

### Limitations and Future Research

Despite contributing to the understanding of students’ subject-specific achievement goal orientation profiles and their emotional outcomes, our study also has some limitations. First, our findings are correlational. In future research, longitudinal designs should be used in order to grasp the developmental dynamics of subject-specific motivation and well-being, preferably supplemented with, for example, register-based information on students’ academic achievement and educational choices. Without longitudinal data, conclusions concerning the adaptiveness of goal orientation profiles are limited to short-term rather than long-term effects; for example, endorsing high multiple goals might enable students to succeed for now, but in the long run, it may become costly. Longitudinal studies starting from an early age are also needed to further explore the origins of achievement goal orientations (e.g., conceptions of ability and intelligence) and possible sources of cross-domain generality *versus* specificity. Furthermore, it would be interesting to complement the design with situation-specific goals, values, and costs as well as with repeated *in situ* measurements of students’ well-being (see Dietrich et al., 2019).

The present study was conducted in the context of Finnish general upper secondary school (i.e., academic track), and thus the generalizability of our findings to students in other less selective educational contexts and in other countries requires further investigation. Our study focused on two key academic domains, mathematics and English, but future research could examine students’ motivational profiles across even a wider array of domains.

One possible weakness of the person-oriented method relates to the decision-making concerning the optimal number of profiles, which always involves researcher discretion. In this study, however, model-based techniques were used, and the fit indices provided by the LPA for facilitating decision-making were rather consistent and pointed quite clearly at the five-class solution, which was also supported by substantive interpretability. Another potential methodological bias related to an approach commonly used in person-oriented studies is that even though LPA permits a person membership in each cluster to a certain degree (i.e., probabilities), still the modal assignment leads to a person being classified in just one cluster, that is, the one with the largest of the posterior probabilities (Pastor et al., 2007). Here, however, modal assignment of persons to clusters was not employed and profile differences in distal outcomes were analyzed instead by using the BCH method (Bakk et al., 2013), and gender was also added as a covariate within the mixture model.

Finally, it should be noted that although the instrument measuring cost seemed to work well in terms of factor structure and validity, the subfacets of cost were rather highly correlated (0.56–0.67 for math, 0.69–0.74 for English). Gaspard and her colleagues also detected high correlations (as high as 0.93–0.97 between effort required and emotional cost within a subject; Gaspard et al., 2017) with this instrument and have, therefore, combined the subfacets of effort and emotional cost in some studies (Gaspard et al., 2017, 2018). Flake et al. (2015) stated that the issue of whether to model cost as a higher-order factor or as separate correlated subdimensions calls for further study but pointed out that it may depend on the research question. In the present study, although our three cost factors were highly correlated, there were some meaningful differential relationships; for example, in math, emotional cost was negatively correlated and effort cost was unrelated with mastery-related orientations, and effort cost was positively related and emotional cost was unrelated with performance-approach orientation. Consequently, employing the separate subdimensions of cost was congruent with our research question and seemed justifiable.

## Conclusion

In the present study, we incorporated achievement goal orientations and costs to enable a more comprehensive understanding of students’ intricate motivational processes, the domain-specificity of achievement motivation, and the implications on well-being. First, our results demonstrated that meaningful patterns of goal orientations in the domains of mathematics and English in the general upper secondary school context emerged (i.e., indifferent, success-oriented, mastery-oriented, avoidance-oriented, and English-oriented, math-avoidant). Mostly, these results echo previous findings, yet these also add to what we know about the cross-domain generality *versus* specificity of achievement goal orientation profiles. On the one hand, the profiles showed more domain-generality than domain-specificity (see also Jansen in de Wal et al., 2016); on the other hand, also a novel goal orientation profile—English-oriented, math-avoidant—was extracted, displaying clear domain-specificity in the achievement goal orientations. The emergence of this profile demonstrates the importance of addressing students’ goals in multiple subjects when studying achievement goal orientation profiles.

Second, our findings showed that the subject-specific achievement goal orientation profiles were associated with both subject-specific perceived cost and more general school engagement and burnout, which might have important practical implications. Conflating negative cost values alongside achievement goal orientations enabled the identification of students experiencing specific combinations of subject-specific motivations and pressures. Overall, mastery-oriented students perceived low costs and showed the most adaptive academic well-being. Based on our findings, we can encourage educational practitioners to create educational environments that foster mastery goals. In turn, avoidance-oriented students were the least engaged, although they did not perceive studying as particularly costly. The extraction of the small group of avoidance-oriented students aiming mainly at effort reduction in all schoolwork stresses the need for teachers to identify such students and attempt to support their engagement and valuing of school. In addition to the rather coherently adaptive (mastery-oriented) and maladaptive (avoidance-oriented) profiles, interesting asynchronous profiles were also found, for example, the profile of success-oriented students, in which not only all achievement goals but also costs were high, which was linked to both positive (engagement) and negative (exhaustion) indices of academic well-being. Regarding practical implications, it is thus important to note that there are many students who seemingly thrive in school but whose success coincides with strain and high cost. Teachers and parents should be aware that when a student aims to excel and demonstrate superiority over others, this sort of performance mode might result in not only academic success but also vulnerability for experiencing pressure, psychological cost, emotional exhaustion, anxiety, and fear of failure (see also Tuominen-Soini et al., 2008, 2011; Jang and Liu, 2012). Furthermore, even though the study-related pressure and the elevated costs would not lead to eroding achievement in the short run, the study-related pressure might influence the students’ educational choices and paths in the long run (see Korhonen et al., 2014; Salmela-Aro and Upadyaya, 2017; Widlund et al., 2020).

In addition to the success-oriented, the English-oriented, math-avoidant students also displayed mixed configurations of costs and well-being indicators as they perceived only studying math as costly, and in terms of academic well-being, they exhibited rather average engagement but slightly elevated burnout. Jiang et al. (2018) suggested that cost has a critical role in determining avoidance motivation and that students displaying high cost can be at risk for inferior math achievement; hence, in practice, reducing cost might be one possible way to promote students’ achievement, persistence, and adjustment. In the present study, those students exhibiting high avoidance tendencies in both subjects (avoidance-oriented) did not display especially high costs. However, the English-oriented, math-avoidant students perceived studying math as emotionally costly, and thus these students are particularly those who might benefit from efforts to reduce students’ cost perceptions in math (for novel cost reduction interventions, see Rosenzweig et al., 2020). Mathematics is a high-stakes subject for many students as good performance in math in general upper secondary school gives a head start in the student selections for higher education in certain fields, thus possibly making studying math especially stressful and highlighting the need for cost reduction.

By simultaneously considering several different motivational indicators (i.e., achievement goal orientations and costs) in key academic domains, a person-oriented approach allowed us to capture students’ multifaceted motivation and to better understand the multiple ways that students utilize motivational resources to support learning, engagement, and well-being (see also Linnenbrink-Garcia and Wormington, 2019).

## Data Availability Statement

The raw data supporting the conclusions of this article will be made available by the authors, without undue reservation.

## Ethics Statement

The studies involving human participants were reviewed and approved by the ethical review board in humanities and social and behavioral sciences of the University of Helsinki, Finland. Written informed consent to participate in this study was provided by the participants’ legal guardian/next of kin.

## Author Contributions

HT collected and analyzed the data, and wrote the first draft of the manuscript. HJ conducted preliminary analyses and contributed to the writing of the manuscript. MN provided support for the data analysis and contributed to the writing of the manuscript. All authors contributed to the article and approved the submitted version.

## Conflict of Interest

The authors declare that the research was conducted in the absence of any commercial or financial relationships that could be construed as a potential conflict of interest.
