# Supplementary material for: Striving for Success but at What Cost? Subject-Specific Achievement Goal Orientation Profiles, Perceived Cost, and Academic Well-Being
Source: Front Psychol. 2020 Sep 29;11:557445. doi: 10.3389/fpsyg.2020.557445 (PMC7550833; doi:10.3389/fpsyg.2020.557445)
Supplement: Supplementary file 1 [file Table_1.pdf]

Supplementary Material

1 Confirmatory Factor Analyses: Factor Loadings and Residual Variances

Table S1: Standardized Factor Loadings and Residual Variances for the Chosen Measurement Models for Achievement Goal Orientations in Mathematics and English

	Factor loadings					
Item	Mastery-intrinsic M / E	Mastery-extrinsic M / E	Performance-approach M / E	Performance-avoidance M / E	Avoidance M / E	Residual variances M / E
MI1	.853 / .810					.272 / .344
MI2	.869 / .881					.245 / .225
MI3	.887 / .806					.214 / .351
ME1		.855 / .776				.269 / .398
ME2		.890 / .810				.208 / .343
ME3		.883 / .855				.220 / .269
PAP1			.680 / .659			.538 / .566
PAP2			.633 / .680			.600 / .537
PAP3			.782 / .729			.389 / .469
PAV1[1]				.580 / .576		.663 / .668
PAV2[1]				.610 / .636		.627 / .595
PAV3				.924 / .944		.146 / .109
AV1					.524 / .533	.725 / .716
AV2					.823 / .893	.322 / .203
AV3					.698 / .617	.513 / .619

Note. M = math; E = English; MI = mastery-intrinsic orientation; ME = mastery-extrinsic orientation; PAP = performance-approach orientation; PAV = performance-avoidance orientation; AV = avoidance orientation.
For math and English, error covariances between one pair of similarly worded items were freed: [1] Items PAV1 "I try to avoid situations in which I may appear dumb or incompetent" and PAV2 "I try to avoid situations in which I may fail or make mistakes".

Table S2: Standardized Factor Loadings and Residual Variances for the Chosen Measurement Models for Perceived Cost in Mathematics and English

	Factor loadings			
Item	Effort required M / E	Emotional cost M / E	Opportunity cost M / E	Residual variances M / E
EFF1	.725 / .771			.474 / .406
EFF2	.872 / .866			.240 / .250
EFF3[1]	– / .869			– / .245
EMO1		.686 / .555		.530 / .692
EMO2		.883 / .879		.221 / .227
EMO3		.834 / .828		.304 / .315
OPP1[2]			.583 / .618	.660 / .619
OPP2[2]			.723 / .766	.478 / .412
OPP3			.842 / .827	.291 / .317

Note. M = math; E = English; EFF = effort required; EMO = emotional cost; OPP = opportunity cost.
For math, one item[1] (EFF3 "Learning mathematics exhausts me") was excluded and, for math and English, error covariances between one pair of similarly worded items were freed: [2] Items OPP1 "I have to give up other activities that I like to be successful at math/English" and OPP2 "I have to give up a lot to do well in math/English".

Table S3: Standardized Factor Loadings and Residual Variances for the Chosen Measurement Model for Academic Well-Being

	Factor loadings				
Item	Engagement	Exhaustion	Cynicism	Inadequacy	Residual variances
ENG1[1]	.769				.409
ENG2[1]	.731				.465
ENG3	.736				.458
ENG4	.853				.273
ENG5	.862				.256
ENG6	.623				.612
ENG7	.808				.348
ENG8	.691				.523
ENG9	.722				.479
EXH1		.570			.675
EXH2		.726			.473
EXH3		.767			.412
EXH4		.690			.524
CYN1			.749		.439
CYN2			.857		.266
CYN3			.760		.423
INA1				.586	.657
INA2				.773	.402
INA3				.641	.589

Note. ENG = schoolwork engagement; EXH = exhaustion; CYN = cynicism; INA = inadequacy.
Error covariances between one pair of successive items measuring engagement were freed: [1] Items ENG1 "At school I am bursting with energy" and ENG2 "I find the schoolwork full of meaning and purpose".
